# Supplementary material for: Relative dispersion ratios following fecal microbiota transplant elucidate principles governing microbial migration dynamics
Source: Nat Commun. 2024 May 24;15:4447. doi: 10.1038/s41467-024-48717-z (PMC11126695; doi:10.1038/s41467-024-48717-z)
Supplement: Supplementary file 1 — Supplementary Information [file 41467_2024_48717_MOESM1_ESM.pdf]

Supplementary information

Supplementary Figures

Supplementary Figures 1 – Community composition and colonization frequency following FMT.

Supplementary Figures 2 – Frequency of dominant partner in various phyla

Supplementary Table

Supplementary Table 1 – FMT datasets analyzed in our study

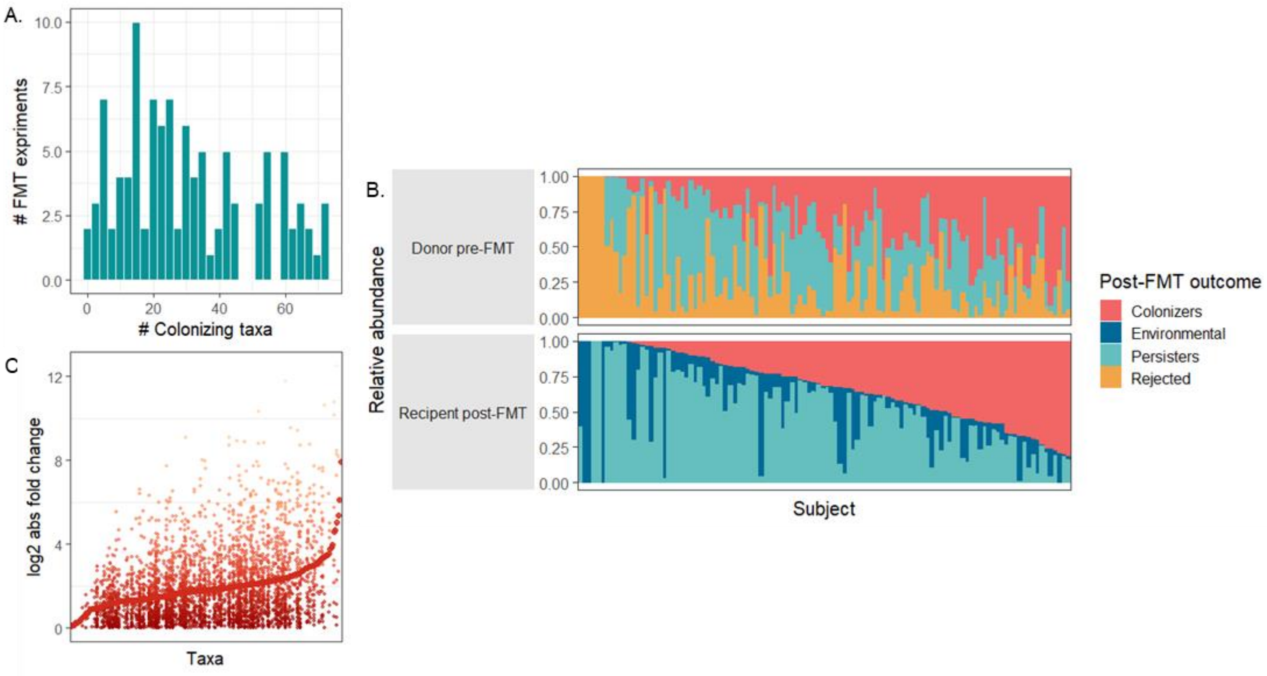

Supplementary Figure 1 – **Community composition and colonization frequency following FMT. A** – Distribution of the number of colonizing taxa observed in each FMT experiment. **B** – Total relative abundance of taxa in the donor pre-FMT and recipient post-FMT samples. Taxa are categorized based on their post-FMT outcomes: colonizing taxa (as described in the main text; highlighted in red), rejected taxa (yellow) that were present in the donor pre-FMT but failed to establish in the recipient post-FMT samples, persistent taxa (light blue) that were identified in both the recipient (pre- and post-FMT) and donor communities, and environmentally acquired taxa (dark blue) that were detected solely in the recipient post-FMT samples. **C** – Colonizing taxa relative abundance fold change (in log2 scale and absolute value) between the donor pre-FMT and the recipient post-FMT environments. Large circles indicate mean fold change for each taxon, and small dots indicate fold change in each FMT experiment.

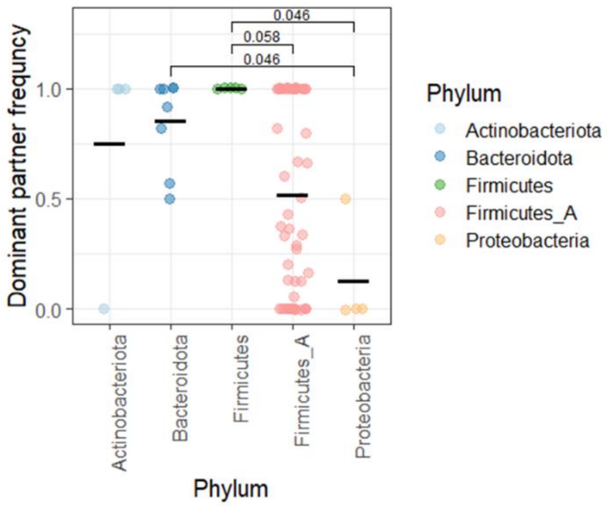

Supplementary Figure 2 – Frequency of dominant partner in various phyla. This figure illustrates the frequency of dominant partnerships based on RDR across different phyla. The y-axis represents the calculated frequency of being the dominant (rather than the minor) partner, while the x-axis displays the various phyla. For clarity and statistical significance, phyla with a sample size of n< 3 were excluded from the figure. Shown are Wilcoxon test FDR corrected p-value.

Supplementary Table 1: FMT datasets analyzed in our study

Study name	Reference	# patients	# samples	# donors	16s region	Dose	Antibiotics preparation	Route of administration	Collection timepoints after FMT (days)	Collection timepoints before FMT (days)
autism_ kang_ 2017	33	18	183	5	V4	High initial dose ($2*10^{12}$ cells) Maintenance dose ($2*10^{9}$ cells)	yes	Oral capsules or colonoscopy	7,14, 28,56, 42,84, 98,112	-14
c_diff_ khanna_ 2017	27	33	123	33	V4	homogenized and diluted 50g of fresh stool	no	colonoscopy	7,28	0
c_diff_ seekatz_ 2014	28	10	30	10	V4	25-50ml of stool suspension	yes	Nasogastric	30	-14
c_diff_ zao_ 2018	29	11	43	11	V3-V4	50g of stool suspension	yes	Nasoduodenal	14,28, 35,42, 70,77, 119,126 (subset)	-5
cancer_ baruch_ 2020	34	10	50	2	V4	Colonoscopy: ~50g stool suspension, Capsules: material from ~15g stool (concentrated)	yes	Colonoscopy and oral capsules	7,31,65	-14
crohn_ sokol_ 2020	30	8	56	7	V3-V4	50-100g of stool suspension	no	Colonoscopy	14,42,70, 98,126, 168	-14
IBD_ goyal_ 2018	31	21	99	21	V4	150g of stool suspension	yes	Colonoscopy	7,30,180	-7
UC_ kump_ 2017	32	13	40	12	V4	50g of stool suspension	yes	Colonoscopy	3,14	-10
Total		**124**	**624**	**100**						
