# Supplementary material for: Relative dispersion ratios following fecal microbiota transplant elucidate principles governing microbial migration dynamics
Source: Nat Commun. 2024 May 24;15:4447. doi: 10.1038/s41467-024-48717-z (PMC11126695; doi:10.1038/s41467-024-48717-z)
Supplement: Supplementary file 3 — Reporting Summary [file 41467_2024_48717_MOESM3_ESM.pdf]

nature portfolio

Corresponding author(s): Elhanan Borenstein

Last updated by author(s): Mar 25, 2024

Reporting Summary

Nature Portfolio wishes to improve the reproducibility of the work that we publish. This form provides structure for consistency and transparency in reporting. For further information on Nature Portfolio policies, see our Editorial Policies and the Editorial Policy Checklist.

Statistics

For all statistical analyses, confirm that the following items are present in the figure legend, table legend, main text, or Methods section.

n/a	Confirmed	
☐	☒	The exact sample size (*n*) for each experimental group/condition, given as a discrete number and unit of measurement
☐	☒	A statement on whether measurements were taken from distinct samples or whether the same sample was measured repeatedly
☐	☒	The statistical test(s) used AND whether they are one- or two-sided<br>*Only common tests should be described solely by name; describe more complex techniques in the Methods section.*
☐	☒	A description of all covariates tested
☐	☒	A description of any assumptions or corrections, such as tests of normality and adjustment for multiple comparisons
☐	☒	A full description of the statistical parameters including central tendency (e.g. means) or other basic estimates (e.g. regression coefficient) AND variation (e.g. standard deviation) or associated estimates of uncertainty (e.g. confidence intervals)
☐	☒	For null hypothesis testing, the test statistic (e.g. *F*, *t*, *r*) with confidence intervals, effect sizes, degrees of freedom and *P* value noted<br>*Give P values as exact values whenever suitable.*
☒	☐	For Bayesian analysis, information on the choice of priors and Markov chain Monte Carlo settings
☒	☐	For hierarchical and complex designs, identification of the appropriate level for tests and full reporting of outcomes
☐	☒	Estimates of effect sizes (e.g. Cohen's *d*, Pearson's *r*), indicating how they were calculated

Our web collection on statistics for biologists contains articles on many of the points above.

Software and code

Policy information about availability of computer code

Data collection	No software and code was used for data collection.
Data analysis	Raw metagenomic data was processed by qiime2 (https://qiime2.org/, version 2019-1), and denoised by DADA2. Similarly, the qiime2 fragment insertion algorithm was used to calculate phylogenetic relationships and PICRUST2 was used to predict functional abundance per ASV. Seed set calculations were conducted using Python code from Levy et al (reference 50) .<br>Data analysis was conducted in R version 4.02 using the packages “tidyveres” (version 2.00, general data handling and cleaning), “tidymodels” (version 0.14, machine learning pipelines), “ranger” (version 0.13.1, RF model), “FastSHAP” (version 0.0.7, SHAP analysis) and ggplot2 (version 3.3.5, visualization).<br><br>Custom analysis code used in this study can be found on GitHub at: https://github.com/borenstein-lab/RDR

For manuscripts utilizing custom algorithms or software that are central to the research but not yet described in published literature, software must be made available to editors and reviewers. We strongly encourage code deposition in a community repository (e.g. GitHub). See the Nature Portfolio guidelines for submitting code & software for further information.

Data

Policy information about availability of data

All manuscripts must include a data availability statement. This statement should provide the following information, where applicable:

- Accession codes, unique identifiers, or web links for publicly available datasets
- A description of any restrictions on data availability
- For clinical datasets or third party data, please ensure that the statement adheres to our policy

In this work, we relied on previously published and publicly available datasets. Specifically, for the FMT meta-analysis we used raw-sequencing data from references 27-34 (these studies are described in detail in Supplementary Table 1). Data from the American Gut Project (AGP) was obtained from the original AGP publication's accompanying figshare (reference 42, https://figshare.com/search?q=:keyword:%20%22American%20Gut%20Project%22). Lastly, we collected results from published co-cultured competition experiments (reference 41).

Research involving human participants, their data, or biological material

Policy information about studies with human participants or human data. See also policy information about sex, gender (identity/presentation), and sexual orientation and race, ethnicity and racism.

Reporting on sex and gender	The analysis of the FMT clinical trials were based on publicly available data as published in references 27-34. As the analysis was conducted on a per-subject basis, sex was not included as a covariate.
Reporting on race, ethnicity, or other socially relevant groupings	Our analysis is limited to publicly available data where race, ethnicity, or other socially relevant groupings were not available.
Population characteristics	We analyzed publicly available data from previously published papers. No new human data has been collected or generated for this study. For a full description of the cohorts used in our research, please refer to the original publications in references (27-34, 42).
Recruitment	We analyzed publicly available data from previously published papers. No new human data has been collected or generated for this study.
Ethics oversight	NA. We analyzed publicly available data from previously published papers. No new human data has been collected or generated for this study.

Note that full information on the approval of the study protocol must also be provided in the manuscript.

Field-specific reporting

Please select the one below that is the best fit for your research. If you are not sure, read the appropriate sections before making your selection.

☒ Life sciences ☐ Behavioural & social sciences ☐ Ecological, evolutionary & environmental sciences

For a reference copy of the document with all sections, see nature.com/documents/nr-reporting-summary-flat.pdf

Life sciences study design

All studies must disclose on these points even when the disclosure is negative.

Sample size	Available data was searched using PubMed and Google Scholar. The FMT meta-analysis was conducted on data from 8 publications and a total of 226 participants (for study-specific statistics see supplementary table 1). As this analysis completely relied on publicly available data, no power analysis or sample size selection was done by the researchers.
Data exclusions	No exclusion was performed.
Replication	To identify differentially dispersing pairs we applied a random effects model that considers multiple recipients post-FMT collection timepoints, as well as donor identity.
Randomization	To predict the composition of the post-FMT community we used a 10-folds cross-validation scheme.
Blinding	Since data was collected, processed, and published initially as part of a previously published work, blinding during data collection is not relevant.

Reporting for specific materials, systems and methods

We require information from authors about some types of materials, experimental systems and methods used in many studies. Here, indicate whether each material, system or method listed is relevant to your study. If you are not sure if a list item applies to your research, read the appropriate section before selecting a response.

Materials & experimental systems

n/a	Involved in the study
☒	☐ Antibodies
☒	☐ Eukaryotic cell lines
☒	☐ Palaeontology and archaeology
☒	☐ Animals and other organisms
☒	☐ Clinical data
☒	☐ Dual use research of concern
☒	☐ Plants

Methods

n/a	Involved in the study
☒	☐ ChIP-seq
☒	☐ Flow cytometry
☒	☐ MRI-based neuroimaging

Plants

Seed stocks	*Report on the source of all seed stocks or other plant material used. If applicable, state the seed stock centre and catalogue number. If plant specimens were collected from the field, describe the collection location, date and sampling procedures.*
Novel plant genotypes	*Describe the methods by which all novel plant genotypes were produced. This includes those generated by transgenic approaches, gene editing, chemical/radiation-based mutagenesis and hybridization. For transgenic lines, describe the transformation method, the number of independent lines analyzed and the generation upon which experiments were performed. For gene-edited lines, describe the editor used, the endogenous sequence targeted for editing, the targeting guide RNA sequence (if applicable) and how the editor was applied.*
Authentication	*Describe any authentication procedures for each seed stock used or novel genotype generated. Describe any experiments used to assess the effect of a mutation and, where applicable, how potential secondary effects (e.g. second site T-DNA insertions, mosiacism, off-target gene editing) were examined.*
